# Clinical applications of invasive spinal cord stimulation in neurological disorders: a narrative review

**DOI:** 10.3389/fneur.2026.1872091

**Published:** 2026-08-26

**Authors:** Mengyu He, Yu Tan, Ze He, Shuqin Yu, Chen Zhang, YouQing Deng

**Affiliations:** 1 Department of Neurology, The Third Affiliated Hospital of Nanchang University (The First Hospital of Nanchang), Nanchang, Jiangxi, China; 2 Shihezi People’s Hospital, The Third Affiliated Hospital of Shihezi University Medical College, Shihezi, China

**Keywords:** chronic neuralgia, clinical application, mechanism of action, neuromodulation, spinal cord stimulation

## Abstract

**Introduction:**

Invasive spinal cord stimulation (SCS) is used in selected patients with refractory chronic neuropathic pain and has also been explored in spinal cord injury, disorders of consciousness, and Parkinson’s disease. However, the strength of evidence differs substantially across indications. This review summarizes the current clinical applications of invasive SCS and distinguishes relatively established pain indications from emerging neurological applications.

**Methods:**

We conducted a structured search of PubMed, Embase, the Cochrane Central Register of Controlled Trials, Web of Science, Wanfang, and China National Knowledge Infrastructure. The search was supplemented by reference tracking for landmark studies and a targeted update of relevant publications from 2024 to 2026.

**Results:**

Randomized evidence is concentrated in chronic pain populations. Conventional SCS has shown benefit in selected patients with failed back surgery syndrome, while 10-kHz SCS produced greater pain reduction than conventional low-frequency stimulation in patients with chronic back and leg pain. Burst stimulation was noninferior to tonic stimulation in the SUNBURST trial and was preferred by most participants. Randomized evidence also supports 10-kHz SCS in refractory painful diabetic neuropathy. Evidence for complex regional pain syndrome suggests short- to intermediate-term pain relief, although long-term benefit remains uncertain. By contrast, applications in diabetic foot complications, spinal cord injury, and disorders of consciousness are supported mainly by small, heterogeneous, or uncontrolled studies. Recent blinded trials have not demonstrated a clear benefit of SCS for Parkinsonian gait impairment.

**Discussion:**

The clinical evidence supporting invasive SCS varies considerably across neurological indications. Its role is comparatively well supported in selected chronic neuropathic pain populations, particularly chronic back and leg pain after spinal surgery and painful diabetic neuropathy. Evidence for non-pain neurological disorders remains limited, and these applications should remain investigational until further controlled studies clarify patient selection, stimulation parameters, durability of response, and safety.

## 1 Introduction

Invasive spinal cord stimulation (SCS) involves the surgical placement of one or more electrodes in the epidural space, with the electrodes connected to an implantable pulse generator. Electrical stimulation delivered through the epidural leads modulates spinal and supraspinal neural circuits and is primarily used in the management of refractory chronic neuropathic pain (1, 2).

Invasive epidural SCS should be distinguished from related but technically and physiologically different forms of electrical stimulation. Transcutaneous spinal cord stimulation delivers electrical current through surface electrodes without surgical implantation. Functional electrical stimulation and neuromuscular electrical stimulation primarily activate peripheral nerves or muscles to facilitate movement. Evidence obtained with these non-invasive or peripheral techniques should therefore not be interpreted as direct evidence for implanted epidural SCS.

The historical basis of SCS is commonly traced to the gate control theory proposed by Melzack and Wall (3). According to this theory, activation of large-diameter myelinated afferent fibers inhibits nociceptive transmission from smaller Aδ and C fibers in the dorsal horn. In 1967, Shealy and colleagues reported the first clinical use of dorsal column stimulation for intractable pain (4). Subsequent observations showed that gate control alone could not fully explain prolonged analgesia after stimulation was discontinued or pain relief achieved with paresthesia-free stimulation paradigms. Current models therefore include modulation of segmental inhibitory circuits, descending pain pathways, neurotransmitter release, neuroinflammatory signaling, and supraspinal network activity (1, 2).

Clinical evidence for SCS has developed primarily in patients with chronic neuropathic pain, including failed back surgery syndrome (FBSS), complex regional pain syndrome (CRPS), and painful diabetic neuropathy (PDN). Invasive epidural stimulation has also been explored in spinal cord injury (SCI), disorders of consciousness (DoC), and Parkinson’s disease (PD). However, evidence for these emerging indications ranges from isolated clinical cases and small uncontrolled cohorts to more recent blinded feasibility studies.

This narrative review summarizes the clinical applications of invasive epidural SCS, distinguishes established pain indications from emerging neurological applications, and critically examines the main limitations of the available evidence.

## 2 Literature search

A structured literature search was conducted using PubMed, Embase, the Cochrane Central Register of Controlled Trials, Web of Science, Wanfang, and China National Knowledge Infrastructure. The initial search covered publications from January 1, 2013, to December 31, 2023. Search terms included combinations of “spinal cord stimulation,” “epidural spinal stimulation,” “failed back surgery syndrome,” “complex regional pain syndrome,” “painful diabetic neuropathy,” “diabetic foot,” “spinal cord injury,” “disorders of consciousness,” “vegetative state,” “minimally conscious state,” and “Parkinson disease.”

English- and Chinese-language clinical trials, observational studies, case series, and relevant reviews were considered. Reference lists of retrieved publications were reviewed to identify landmark studies published before 2013. To address recent developments, a targeted PubMed update was conducted for relevant clinical studies and consensus recommendations published between January 2024 and July 2026.

Animal studies were considered only when they provided relevant mechanistic or translational context and were not treated as evidence of clinical efficacy. Non-invasive transcutaneous SCS, transcutaneous electrical nerve stimulation, functional electrical stimulation, peripheral nerve stimulation, and isolated muscle stimulation were excluded from the principal clinical synthesis unless discussed specifically to clarify differences among stimulation modalities.

Because this article was designed as a narrative review rather than a systematic review, no meta-analysis or formal pooled risk-of-bias assessment was performed. The evidence was interpreted according to study design, sample size, presence of a comparator or sham condition, follow-up duration, and consistency of the reported findings.

## 3 Clinical applications

The clinical evidence for implanted SCS differs considerably across indications. Randomized trials are available primarily for chronic neuropathic pain, whereas studies in SCI, DoC, and PD are more heterogeneous and frequently involve small samples or uncontrolled designs. Representative clinical studies across established and emerging indications are summarized in Table 1.

**Table 1:** Representative clinical studies of invasive spinal cord stimulation across established and emerging indications.

Indication	First author, year	Study design	Sample size	SCS paradigm and comparator	Main findings	Main limitations
Failed back surgery syndrome	Kumar, 2007; 2008 (5, 6)	Multicenter randomized controlled trial with extended follow-up	100	Conventional tonic SCS plus conventional medical management (CMM) vs. CMM alone	At 6 months, ≥50% leg-pain relief was achieved by 48% of patients assigned to SCS and 9% assigned to CMM alone. Follow-up suggested sustained benefit in a proportion of implanted patients.	Open-label treatment; substantial crossover complicated longer-term between-group comparisons.
Complex regional pain syndrome type I	Kemler, 2000 (7)	Randomized controlled trial	54	Conventional SCS plus physical therapy vs. physical therapy alone	SCS plus physical therapy produced greater short-term pain reduction than physical therapy alone in selected patients.	Small sample size; only selected patients were suitable for stimulation; long-term between-group benefit diminished.
Complex regional pain syndrome type I	Forouzanfar, 2004 (8)	Prospective cohort study	36	Cervical vs. lumbar implanted SCS	Pain intensity and health status improved after implantation, with no clear difference between cervical and lumbar SCS.	Non-randomized comparison; technical complications were frequent; no untreated control group.
Chronic back and leg pain	Kapural, 2015; 2016 (9, 10)	Multicenter randomized controlled trial with extended follow-up	198	Paresthesia-free 10-kHz SCS vs. conventional low-frequency tonic SCS	Ten-kilohertz SCS produced higher responder rates for back and leg pain, with benefit maintained in a substantial proportion of patients during extended follow-up.	No sham control; most, but not all, participants had undergone previous spinal surgery; paresthesia may have affected blinding.
FBSS or radiculopathy	Deer, 2018 (11)	Prospective multicenter randomized crossover trial	100	Burst SCS vs. tonic SCS	Burst stimulation was noninferior to tonic stimulation for overall pain intensity, and 70.8% of participants preferred the burst waveform.	Participants had already responded to tonic SCS during trial stimulation, resulting in an enriched study population.
Painful diabetic neuropathy	Petersen, 2021; 2023 (12, 13)	Multicenter randomized controlled trial with long-term follow-up	216	10-kHz SCS plus CMM vs. CMM alone	The primary composite endpoint was achieved by 79% of patients assigned to SCS plus CMM and 5% assigned to CMM alone. Pain relief and neurological examination improvements were maintained in many implanted patients at 24 months.	Open-label design; crossover was permitted after 6 months; long-term findings were based mainly on implanted participants rather than a continuing randomized comparison.
Diabetic foot complications	Zhou and Bao, 2023 (14)	Clinical evaluation study	32	Implanted low-frequency epidural SCS	Treatment was associated with reductions in pain and improvements in lower-limb perfusion and selected neurological measures.	Small, single-center study; limited comparator information; vascular treatment and other concomitant interventions may have affected outcomes.
Spinal cord injury	Harkema, 2011 (15)	Case study	1	Lumbosacral epidural stimulation combined with activity-based training	Epidural stimulation enabled voluntary movement, standing, and assisted stepping in a participant with motor-complete paraplegia.	Single participant; intensive rehabilitation was administered concurrently; findings cannot establish general clinical efficacy.
Spinal cord injury	Wagner, 2018 (16)	Proof-of-concept clinical study	3	Targeted spatiotemporal epidural stimulation combined with rehabilitation	Stimulation facilitated voluntary control of paralyzed muscles and supported overground walking during rehabilitation.	Very small sample; no control group; stimulation was combined with intensive rehabilitation.
Spinal cord injury	Gill, 2018 (17)	Case study	1	Lumbosacral epidural stimulation combined with multimodal rehabilitation	The participant achieved independent stepping during stimulation after complete paraplegia.	Single participant; intensive rehabilitation and stimulation were combined; generalizability is very limited.
Spinal cord injury	Angeli, 2018 (18)	Prospective case series	4	Customized epidural SCS combined with intensive locomotor training	Two of four participants recovered overground walking with stimulation, and the other participants achieved standing or stepping-related gains.	Very small, uncontrolled study; treatment combined SCS with intensive rehabilitation.
Spinal cord injury	Rowald, 2022 (19)	Prospective feasibility case series	3	Activity-specific epidural stimulation using personalized electrode configurations	Activity-specific stimulation enabled standing, walking, cycling, swimming, and trunk control during active stimulation.	Very small, uncontrolled study; outcomes during active stimulation do not necessarily indicate permanent neurological recovery.
Spinal cord injury	Angeli, 2025 (20)	Prospective cohort analysis	30	Epidural SCS combined with activity-based standing training	All participants achieved some lower-limb independent extension during stimulation; 16 achieved unassisted hip extension with bilateral knee and trunk extension.	SCS was combined with 80–160 rehabilitation sessions; the independent effect of stimulation could not be isolated.
Disorders of consciousness	Yamamoto, 2013 (21)	Clinical series/institutional experience	10 SCS-treated MCS patients	Cervical epidural SCS	Clinical and electrophysiological outcomes were reported in selected MCS patients treated with cervical SCS.	Highly selected patients; no sham control; findings were reported alongside DBS experience and require cautious interpretation.
Disorders of consciousness	Xia, 2021 (22)	Clinical case series	24	Cervical epidural SCS	Changes in cortical electrophysiological components were observed after stimulation in patients with chronic disorders of consciousness.	Small, uncontrolled, single-center study; clinical significance of electrophysiological changes remains uncertain.
Disorders of consciousness	Yuan, 2018 (23)	Retrospective clinical cohort	30	Cervical epidural SCS	Behavioral arousal and improvements in consciousness-related assessments were reported after stimulation.	Retrospective, uncontrolled study; limited methodological detail and potential selection bias.
Disorders of consciousness	Yamamoto, 2017 (24)	Uncontrolled clinical series	31 (21 VS; 10 MCS)	Cervical epidural SCS at C2–C4, typically 5 Hz	Behavioral, consciousness-related, motor, and cerebral-perfusion changes were reported in selected patients.	Highly selected participants; no control or sham group; heterogeneous diagnoses and potential contribution of natural recovery.
Disorders of consciousness	Sun, 2024 (26)	Uncontrolled clinical study	27	Cervical epidural SCS	Improvements in consciousness assessments, EEG spectral patterns, and P300 measures were reported after stimulation.	No sham control; heterogeneous etiologies and disease durations; natural recovery could not be excluded.
Parkinson’s disease-related gait dysfunction	Samotus, 2018 (28)	Open-label pilot study	5	Thoracic epidural SCS	Improvements were reported in selected gait and freezing-of-gait measures.	Very small sample; no sham or control condition; placebo effects and concurrent treatment cannot be excluded.
Parkinson’s disease	Carra, 2025 (29)	Randomized crossover double-blind study	8 implanted; 7 completed	Thoracic epidural SCS with blinded stimulation conditions	SCS did not significantly improve gait or overall motor symptoms; the study was terminated for futility.	Small sample; early termination; only seven participants completed the double-blind evaluation.
Parkinson’s disease	Terkelsen, 2026 (30)	Double-blind randomized feasibility trial with open-label extension	14 assessed; 12 randomized	MicroBurst SCS vs. sham for 6 months, followed by an open-label extension	The primary gait outcome did not differ significantly from sham at 6 months; selected lower-body rigidity and bradykinesia measures improved during longer follow-up.	Small feasibility trial; not powered to establish efficacy; protocol deviations and attrition limit interpretation.

### 3.1 Established and relatively well-supported pain indications

#### 3.1.1 Failed back surgery syndrome and chronic back and leg pain

FBSS is one of the most extensively studied indications for invasive SCS. In the PROCESS trial, Kumar et al. (5) compared conventional medical management alone with SCS plus conventional medical management in 100 patients with FBSS. At 6 months, at least 50% leg-pain relief was achieved by 48% of patients assigned to SCS and 9% of patients assigned to conventional medical management alone. Longer-term follow-up suggested that clinically meaningful pain relief was maintained in a proportion of implanted patients, although interpretation of between-group differences was complicated by treatment crossover (6).

#### 3.1.2 Complex regional pain syndrome

Kemler et al. (7) reported that SCS combined with physical therapy produced greater short-term pain reduction than physical therapy alone in selected patients with CRPS type I. However, the between-group difference diminished during long-term follow-up. These findings suggest that SCS may provide short- to intermediate-term pain relief in carefully selected patients, whereas the durability of benefit remains uncertain.

Forouzanfar et al. (8) compared cervical and lumbar electrode placement in patients with CRPS and reported broadly comparable clinical outcomes between the two anatomical approaches. This study evaluated the effect of electrode location and should not be described as a comparison between SCS and physical therapy.

#### 3.1.3 High-frequency and burst stimulation

Conventional tonic SCS usually produces paresthesia over the painful region and requires adequate overlap between the paresthesia and pain distributions. Ten-kilohertz SCS was developed as a paresthesia-free alternative.

The SENZA-RCT compared paresthesia-free 10-kHz SCS with conventional low-frequency SCS in 198 patients with chronic back and leg pain, most of whom had previously undergone spinal surgery (9, 10). Ten-kilohertz SCS produced higher responder rates for both back and leg pain, with benefit maintained in a substantial proportion of patients during extended follow-up. However, because not all participants met a formal definition of FBSS, the entire SENZA-RCT population should not be described as having FBSS.

The SUNBURST trial compared burst stimulation with tonic stimulation in patients who had responded to an SCS trial. Burst stimulation met the prespecified noninferiority endpoint for overall pain intensity, and 70.8% of participants preferred the burst waveform (11). These findings support burst stimulation as an alternative waveform but should not be interpreted as demonstrating universal superiority over tonic stimulation.

#### 3.1.4 Painful diabetic neuropathy

The SENZA-PDN trial evaluated 10-kHz SCS plus conventional medical management against conventional medical management alone in 216 patients with refractory PDN (12). At the primary assessment, 79% of patients assigned to 10-kHz SCS met the composite endpoint of at least 50% pain relief without worsening neurological deficits, compared with 5% of patients receiving conventional medical management alone. Improvements on neurological examination were also reported more frequently in the SCS group.

Follow-up of implanted patients suggested that pain relief and neurological examination improvements were maintained in many patients at 24 months (13). These findings provide evidence for durable benefit among treated patients. However, because crossover to SCS was permitted after 6 months, the longer-term results should not be presented as a continuing randomized comparison between SCS and conventional medical management.

#### 3.1.5 Diabetic foot complications

Evidence for diabetic foot complications should be distinguished from evidence for PDN. Zhou and Bao reported that implanted low-frequency SCS was associated with reduced pain, improved lower-limb perfusion, and improvements in selected neurological measures in patients with diabetic foot complications (14). However, the small sample size, limited comparator information, and potential contribution of vascular treatment, wound care, and other concomitant interventions restrict the strength of the conclusions.

### 3.2 Emerging and exploratory neurological indications

#### 3.2.1 Spinal cord injury

Epidural SCS has been investigated as a means of increasing the excitability of residual spinal sensorimotor networks after SCI. Early proof-of-concept studies reported voluntary movement, standing, assisted stepping, or overground walking during stimulation in patients with clinically motor-complete injuries (15–18). Personalized, activity-specific stimulation strategies have subsequently enabled selected participants to perform standing, walking, cycling, swimming, and trunk movements during active stimulation (19).

These findings established the feasibility of using epidural stimulation to facilitate motor output after SCI. Nevertheless, several influential studies involved only one to four participants and combined SCS with prolonged, intensive activity-based rehabilitation. The independent contribution of stimulation is therefore difficult to determine, and functional performance during active stimulation should not automatically be interpreted as permanent neurological recovery.

A larger 2025 study evaluated 30 individuals with chronic cervical SCI who underwent activity-based standing training combined with targeted epidural stimulation. All participants achieved some degree of independent lower-limb extension during stimulation, and 16 achieved unassisted hip extension while maintaining bilateral knee and trunk extension. However, the intervention combined stimulation with 80–160 structured training sessions, meaning that the effects of stimulation and rehabilitation could not be fully separated (20).

Overall, epidural stimulation may facilitate selected motor functions when combined with intensive rehabilitation, but evidence for durable, stimulation-independent functional recovery remains limited.

#### 3.2.2 Disorders of consciousness

Cervical epidural SCS has been explored in patients with vegetative state, unresponsive wakefulness syndrome, and minimally conscious state (21–25). Small uncontrolled studies have reported behavioral changes, increased regional cerebral perfusion, altered cortical electrophysiological activity, and improvements in consciousness-related scales after stimulation.

A 2024 study of 27 patients with prolonged DoC evaluated behavioral status, scalp electroencephalography, and P300 event-related potentials before and after SCS. Postoperative changes in EEG spectral patterns and P300 measures were reported alongside improvements in consciousness assessments (26). Although these findings add objective electrophysiological data, the absence of a sham-treated control group limits causal interpretation.

Interpretation of the broader DoC literature remains difficult because of heterogeneous brain-injury etiologies, differences in time since injury, variable diagnostic criteria, inconsistent definitions of response, and the absence of adequately powered sham-controlled trials. Natural recovery, rehabilitation, and changes in concomitant treatment may also contribute to observed improvements. Cervical SCS should therefore remain an investigational intervention for DoC.

#### 3.2.3 Parkinson’s disease-related gait impairment

Preclinical work by Fuentes et al. (27) showed that thoracic SCS improved locomotor activity and altered pathological neural oscillations in rodent models of PD. This study provides a biological rationale for clinical investigation but should not be classified as a human clinical trial.

In an early open-label study, Samotus et al. (28) reported improvements in selected gait and freezing-of-gait measures in five patients with advanced PD. However, the absence of a control condition, very small sample size, and potential placebo effects substantially limited interpretation.

More recent controlled studies have produced less favorable results. In 2025, Carra et al. (29) reported a randomized, crossover, double-blind evaluation in which SCS did not significantly improve gait or overall motor symptoms despite extensive adjustment of stimulation parameters.

The 2026 STEP-PD study enrolled 14 participants for clinical and imaging assessments, of whom 12 were randomized to receive MicroBurst stimulation or sham stimulation for 6 months, followed by an open-label extension. The procedure was feasible, but active stimulation did not significantly improve the primary postural instability and gait outcome compared with sham treatment at 6 months. Improvements in selected lower-body bradykinesia and rigidity measures were observed during longer follow-up, but the study was not powered to establish clinical efficacy (30).

Taken together, current evidence for SCS in PD is mixed. Early uncontrolled studies suggested possible benefit, whereas recent blinded investigations have not demonstrated a clear effect on gait or overall motor function. SCS should therefore not be regarded as an established treatment for Parkinsonian gait impairment.

## 4 Technical and safety considerations

Implantation of an SCS system is associated with procedure- and device-related risks, including infection, lead migration, lead fracture, epidural bleeding, neurological injury, hardware malfunction, loss of therapeutic effect, and the need for revision or explantation. The frequency of these complications varies according to implantation technique, electrode type, patient characteristics, follow-up duration, and the definitions used in individual studies.

Recent Neurostimulation Appropriateness Consensus Committee recommendations emphasize careful patient selection, perioperative infection prevention, anticoagulation management, appropriate surgical technique, recognition of neurological complications, and systematic management of hardware-related problems (31).

Long-term management should also include reassessment of lead position, programming, changes in the underlying disease, psychological factors, and potentially correctable causes of reduced efficacy before device removal is considered (32). These issues are particularly relevant in SCI and DoC, in which repeated neuroimaging, electrophysiological assessment, and long-term rehabilitation may be required.

Specific complication percentages should not be presented unless the source population, implantation technique, denominator, and duration of follow-up are clearly stated.

## 5 Discussion

The available evidence for invasive SCS differs substantially across neurological indications. Randomized studies support its use in selected patients with chronic back and leg pain after spinal surgery (5, 6, 9, 10) and refractory PDN (12, 13). Evidence for CRPS is based on fewer randomized data and suggests that early analgesic benefit may diminish during long-term follow-up (7, 8).

Comparisons among stimulation waveforms also require careful interpretation. Ten-kilohertz SCS produced greater pain reduction than conventional low-frequency stimulation in the SENZA-RCT (8–10). In contrast, the SUNBURST trial primarily established the noninferiority of burst stimulation and documented a strong preference for the burst waveform among participants who had already responded to an SCS trial (11). These findings should not be combined into a general statement that both 10-kHz and burst stimulation are uniformly superior to conventional tonic SCS.

Evidence for diabetic foot complications (14), SCI (15–20), DoC (21–26), and PD (28–30) is substantially less certain. Studies in diabetic foot complications frequently include concomitant vascular and wound-care interventions. Most SCI studies combine stimulation with intensive rehabilitation, making it difficult to isolate the effect of SCS. DoC studies remain vulnerable to natural recovery, diagnostic misclassification, and the absence of sham control. In PD, recent blinded studies have not confirmed the benefits suggested by earlier uncontrolled reports.

The therapeutic objective of SCS also differs across indications. In chronic neuropathic pain, stimulation is intended primarily to reduce pain intensity and pain-related disability. In SCI, DoC, and PD, stimulation is used in an attempt to modulate residual motor, autonomic, or consciousness-related networks. Improvement during active stimulation does not necessarily indicate permanent restoration of neurological function.

Several methodological issues limit comparison across studies. Diagnostic definitions and eligibility criteria vary, particularly in FBSS, diabetic foot complications, and DoC. Stimulation protocols differ in electrode position, frequency, pulse width, amplitude, waveform, and daily duration. In addition, many emerging applications use multidimensional treatment programs in which SCS is combined with rehabilitation, medication adjustment, or other interventions.

This review also has limitations. Although several English- and Chinese-language databases were considered and recent studies were added through a targeted update, the review was not designed as a formal systematic evidence synthesis. A quantitative meta-analysis and pooled risk-of-bias assessment were therefore not performed. Studies published in other languages may have been missed, and publication bias is likely in emerging indications because favorable cases may be more likely to be reported than unsuccessful interventions.

## 6 Conclusion

Invasive SCS is supported by randomized evidence for selected patients with refractory chronic neuropathic pain, particularly chronic back and leg pain after spinal surgery and painful diabetic neuropathy. Ten-kilohertz SCS has shown greater pain reduction than conventional low-frequency stimulation in patients with chronic back and leg pain, whereas burst stimulation has demonstrated noninferiority and greater patient preference in selected SCS responders.

Evidence for CRPS suggests short- to intermediate-term pain relief, although long-term benefit is less certain. Evidence for diabetic foot complications, SCI, and DoC remains limited by small samples, heterogeneous methods, and the frequent absence of control groups. Recent blinded trials have not demonstrated a clear benefit of SCS for Parkinsonian gait impairment. These neurological applications should remain investigational until larger controlled studies clarify patient selection, stimulation parameters, clinically meaningful outcomes, durability of response, and procedure-related risks.

## Data Availability

The original contributions presented in the study are included in the article/supplementary material, further inquiries can be directed to the corresponding authors.
